# Trajectory of serial neutrophil-to-lymphocyte ratio predicts neurological outcome after out-of-hospital cardiac arrest

**DOI:** 10.1016/j.resplu.2026.101337

**Published:** 2026-04-21

**Authors:** Kyungman Cha, Sang Hoon Oh, Jee Yong Lim

**Affiliations:** a Department of Emergency Medicine, Suwon St. Vincent Hospital, College of Medicine, The Catholic University of Korea, 93 Jungbu-daero, Paldal-gu, Suwon-si, Gyeonggi-do 16247, the Republic of Korea; b Department of Emergency Medicine, Seoul St. Mary’s Hospital, College of Medicine, The Catholic University of Korea, 222 Banpo-daero, Seocho-gu, Seoul 06591, the Republic of Korea; c International Healthcare Center, Seoul St. Mary’s Hospital, College of Medicine, The Catholic University of Korea, 222 Banpo-daero, Seocho-gu, Seoul 06591, the Republic of Korea

**Keywords:** Cardiac arrest, Neutrophil-to-lymphocyte ratio, Targeted temperature management, Neuroprognostication, Inflammatory biomarker, Lymphocyte recovery

## Abstract

**Aim:**

To evaluate whether serial neutrophil-to-lymphocyte ratio (NLR) trajectory patterns and individual time-point NLR values predict neurological outcome after out-of-hospital cardiac arrest (OHCA).

**Methods:**

This retrospective study analyzed 414 comatose OHCA survivors treated with targeted temperature management at a tertiary center (2009–2024). NLR was calculated at 0, 24, 48, and 72 h after return of spontaneous circulation. The primary outcome was 6-month Cerebral Performance Category dichotomized as good (1–2) or poor (3–5). Patients with complete serial data (*n* = 277) were classified into trajectory groups. A linear mixed-effects model tested group-by-time interaction, and DeLong tests compared discriminative performance across time points.

**Results:**

Among 414 patients, 131 (31.6%) achieved good outcomes. NLR did not differ between groups at 0 or 24 h but diverged at 72 h (median 6.0 vs 13.9; *P* < 0.001; area under the curve 0.785), driven by failure of absolute lymphocyte count recovery in poor-outcome patients (median 0.86 vs 1.28 × 10^3^/µL; *P* < 0.001). Trajectory analysis identified early resolution (60.6% good outcome), sustained elevation (31.9%), and late rise (10.5%; adjusted odds ratio 22.6; *P* < 0.001). Log-transformed NLR at 72 h was independently associated with outcome (odds ratio 5.13; 95% confidence interval 2.88–9.14), and its addition to the clinical model significantly improved discrimination (area under the curve from 0.824 to 0.891; DeLong *P* = 0.001). In a combined model with neuron-specific enolase at 48 h, NLR at 72 h showed a borderline independent association (*P* = 0.050).

**Conclusions:**

Failure of NLR to resolve by 72 h was associated with poor neurological outcome in this cohort. As NLR is derived from routine blood counts at no additional cost, it may serve as an accessible complement to specialized prognostic biomarkers, particularly in settings where neuron-specific enolase is unavailable. These findings require prospective multicenter validation.

## Introduction

Patients resuscitated from out-of-hospital cardiac arrest (OHCA) face a complex pathophysiological cascade known as post-cardiac arrest syndrome, characterized by whole-body ischemia–reperfusion injury, myocardial dysfunction, and a systemic inflammatory response that shares features with sepsis.^1^, ^2^, ^3^ The magnitude of this inflammatory response has been consistently linked to the severity of organ dysfunction and neurological injury.^4^, ^5^ Despite advances in targeted temperature management (TTM) and post-resuscitation care, reliable prognostication of neurological outcomes remains a clinical challenge, particularly during the early days after return of spontaneous circulation (ROSC).^6^, ^7^

Current prognostication strategies rely on a multimodal approach incorporating clinical examination, electrophysiology, neuroimaging, and serum biomarkers such as neuron-specific enolase (NSE).^7^, ^8^ While these tools offer high specificity, they require specialized equipment or assays that may not be universally available. There is a recognized need for simple, cost-free, and widely accessible biomarkers that can complement established prognostic tools.^9^

The neutrophil-to-lymphocyte ratio (NLR), calculated from routine complete blood counts, has been studied as a prognostic indicator in various critical illnesses including sepsis, stroke, and acute coronary syndromes.^10^, ^11^, ^12^ In the context of cardiac arrest, several studies have examined NLR at single time points and reported associations with mortality and neurological outcomes.^13^, ^14^, ^15^, ^16^ However, these studies have generally treated NLR as a static measurement, without exploring the dynamic changes in the post-arrest inflammatory response.

The trajectory of NLR over time may carry different prognostic information compared with any single measurement. Following cardiac arrest, an initial stress response drives neutrophilia and lymphopenia in most patients regardless of outcome. A single NLR measurement captures this response at one moment but cannot distinguish whether the elevation is transient or persistent. By contrast, serial measurements over the first 72 h can track whether the immune response resolves or fails to recover—a distinction that may be more informative for prognosis. To our knowledge, serial NLR trajectory patterns in OHCA survivors have not been systematically characterized in relation to neurological prognosis.

We therefore aimed to (1) compare the discriminative performance of NLR at individual time points (0, 24, 48, and 72 h) for predicting neurological outcome, (2) describe the temporal dynamics of NLR over the first 72 h after ROSC in OHCA patients treated with TTM, (3) identify distinct NLR trajectory patterns, and (4) evaluate the prognostic value of these trajectory patterns for 6-month neurological outcomes.

## Methods

### Study design and setting

This single-center retrospective cohort study analyzed prospectively collected registry data from consecutive OHCA patients treated with TTM at Seoul St. Mary’s Hospital, a tertiary referral center, between January 2009 and December 2024. The study was conducted according to the guidelines of the Declaration of Helsinki and approved by the Institutional Review Board of the Catholic University of Korea, Seoul St. Mary’s Hospital (KC23RISI0264; approved 21 April 2023), which waived the requirement for informed consent given the retrospective nature of the analysis.

### Patients

Adult patients (≥18 years) who experienced non-traumatic OHCA, achieved sustained ROSC, and were treated with TTM were eligible for inclusion. Patients were excluded if they had a pre-arrest Cerebral Performance Category (CPC) score of 3–5, died within 24 h of admission without prognostic evaluation, had a known hematological malignancy (e.g., leukemia, lymphoma, myelodysplastic syndrome) or were receiving immunosuppressive therapy that could directly affect leukocyte differential counts, or had no follow-up data available at 6 months.

### Post-resuscitation care and TTM protocol

All patients received post-cardiac arrest care in accordance with contemporary guidelines. TTM was initiated as soon as possible after ROSC. Over the study period, the target temperature evolved: 33°C was used exclusively from 2009 to 2013. From 2014 onward, after publication of the TTM trial, both 33°C and 36°C were used at the discretion of the treating physician. The proportion of patients managed at 36°C increased modestly after 2020 following the TTM2 trial, but 33°C remained the predominant target throughout the study period (377/414 [91.1%] at 33°C, 27 [6.5%] at 36°C). The target temperature was maintained for 24 h, followed by controlled rewarming at 0.25°C/h. Post-resuscitation care bundles, including the use of early coronary angiography for suspected cardiac etiology, were adopted progressively during the study period. Neurological prognostication was performed using a multimodal approach at 72 h or later, incorporating clinical examination, somatosensory evoked potentials, electroencephalography, brain computed tomography, and magnetic resonance imaging where available. Withdrawal of life-sustaining treatment was not practiced during the study period, eliminating the risk of self-fulfilling prophecy.

### Data collection

Demographic and clinical variables were extracted from the prospective registry, including age, sex, comorbidities, arrest characteristics (witnessed status, bystander cardiopulmonary resuscitation [CPR], initial rhythm, presumed etiology), time from collapse to ROSC, and TTM protocol details. Complete blood counts with differential, including absolute and percentage neutrophil and lymphocyte counts, were obtained as part of routine clinical care at presentation (0 h) and at 24, 48, and 72 h after ROSC.

### NLR calculation and trajectory classification

NLR was calculated as the ratio of neutrophil percentage to lymphocyte percentage at each time point. For the trajectory analysis, patients with NLR available at all four time points (0, 24, 48, and 72 h) were included. Trajectory patterns were classified based on NLR dynamics relative to the 24–48 h peak: early resolution was defined as NLR declining to less than 50% of the 24–48 h peak value by 72 h; sustained elevation was defined as NLR peaking before 72 h but not declining to less than 50% of the peak by 72 h; and late rise was defined as NLR continuing to rise with the highest value occurring at 72 h. The 50% decline threshold was chosen a priori as it represents the midpoint between full persistence and complete resolution and is analogous to thresholds used in previous biomarker trajectory studies in critical care; sensitivity analyses using 25% and 75% thresholds were performed to assess robustness.

We chose this rule-based classification over data-driven approaches such as group-based trajectory modeling (GBTM) or latent class growth analysis (LCGA) for several reasons. First, a clinically intuitive classification scheme—based on whether NLR resolves, persists, or worsens—allows for straightforward bedside interpretation without requiring specialized software. Second, the three trajectory patterns map onto biologically plausible post-arrest immune phenotypes (resolution, persistence, and escalation of systemic inflammation). Third, with 277 patients and four time points, the sample size may be insufficient for reliable latent class estimation, which typically requires larger cohorts to identify stable trajectory classes. Nevertheless, we acknowledge that GBTM or LCGA may be better suited for larger multicenter datasets and could provide additional insights into the heterogeneity of post-arrest NLR trajectories.

### Outcome measures

The primary outcome was neurological status at 6 months, assessed using the CPC scale and dichotomized as good (CPC 1–2) or poor (CPC 3–5).

### Statistical analysis

Continuous variables are presented as median (interquartile range [IQR]) and compared using the Mann–Whitney *U* test. Categorical variables are reported as frequencies (percentages) and compared using the chi-square test or Fisher’s exact test as appropriate. The discriminative performance of NLR at each time point was assessed using the area under the receiver operating characteristic curve (AUC). Pairwise comparisons of AUCs between time points and between nested prediction models were performed using the DeLong test. Optimal cut-off values were determined by the Youden index.

To examine overall temporal changes in NLR and test whether the trajectory differed between outcome groups, we used two complementary approaches: (1) the Friedman test (a non-parametric repeated-measures analysis) within each outcome group, applied to patients with complete data at all four time points; and (2) a linear mixed-effects model with log-transformed NLR as the dependent variable, time and outcome group as fixed effects with their interaction term, and a random intercept for each patient. The group-by-time interaction term tested whether the temporal NLR trajectory differed between good- and poor-outcome patients.

Multivariable logistic regression was performed to evaluate the independent association between log-transformed NLR at 72 h and neurological outcome, adjusting for age, initial shockable rhythm, witnessed arrest, bystander CPR, and time to ROSC. Log-transformation was applied because NLR values were right-skewed (Shapiro–Wilk *P* < 0.001); this transformation is standard for ratio-based biomarkers and has been used in prior NLR studies in critical care.^10^, ^14^ The incremental discriminative value of adding NLR 72 h was assessed by comparing AUCs (DeLong test) and by calculating the continuous net reclassification improvement (NRI) and integrated discrimination improvement (IDI) with 2000-iteration bootstrap confidence intervals. Odds ratios with 95% confidence intervals were calculated for trajectory groups using early resolution as the reference category. Multivariable logistic regression adjusting for the same covariates was also performed to obtain adjusted odds ratios for trajectory groups.

Analyses at each time point used all available data at that time point (complete case approach). Multiple imputation was not performed because NLR missingness was not random but was associated with clinical severity (patients who died or deteriorated early were less likely to have 72 h blood draws), violating the missing-at-random assumption required for standard imputation methods. Instead, we compared baseline characteristics and 6-month outcomes between patients with and without complete four-time-point data to characterize the direction and magnitude of any selection bias. The robustness of the trajectory classification was assessed by repeating the analysis using 25% and 75% decline thresholds in addition to the primary 50% threshold. A sensitivity analysis stratifying by TTM target temperature was also performed. A two-sided *P* value < 0.05 was considered statistically significant. All analyses were performed using Python 3.11 with SciPy 1.11 and scikit-learn 1.7.

## Results

### Study population

During the study period, 419 OHCA patients were treated with TTM. After excluding 5 patients without 6-month outcome data, 414 patients were included in the analysis. The median age was 56 years (IQR 42–67), 298 (72.0%) were male, and 245 (59.2%) had a cardiac etiology. Good neurological outcomes (CPC 1–2) at 6 months were achieved by 131 patients (31.6%), while 283 (68.4%) had poor outcomes (CPC 3–5). Patients with good outcomes were younger (median 51 vs 58 years; *P* < 0.001) and more frequently had a shockable rhythm (84.7% vs 41.0%; *P* < 0.001), cardiac etiology (90.1% vs 44.9%; *P* < 0.001), and witnessed arrest (80.9% vs 62.2%; *P* < 0.001). Baseline characteristics are presented in Table 1.

**Table 1:** Baseline characteristics of the study population.

**Variable**	**Good outcome (*n* = 131)**	**Poor outcome (*n* = 283)**	***P* value**
Age, years, median (IQR)	51 (39–61)	58 (46–72)	<0.001
Male sex, *n* (%)	100 (76.3)	198 (70.0)	0.221
Cardiac etiology, *n* (%)	118 (90.1)	127 (44.9)	<0.001
Shockable rhythm, *n* (%)	111 (84.7)	116 (41.0)	<0.001
Witnessed arrest, *n* (%)	106 (80.9)	176 (62.2)	<0.001
Bystander CPR, *n* (%)	89 (67.9)	162 (57.2)	0.050
Time to ROSC, min, median (IQR)	2 (0–5)	5 (0–10)	<0.001
Hypertension, *n* (%)	32 (24.4)	101 (35.7)	0.030
Diabetes mellitus, *n* (%)	14 (10.7)	82 (29.0)	<0.001
Prior AMI, *n* (%)	10 (7.6)	17 (6.0)	0.682
CHF, *n* (%)	4 (3.1)	7 (2.5)	0.990
Prior stroke, *n* (%)	0 (0.0)	10 (3.5)	0.067
NLR at 0 h, median (IQR)	1.22 (0.55–3.23)	1.61 (0.78–3.66)	0.067
WBC at 0 h, ×10^3^/µL	13.3 (10.5–17.6)	12.6 (9.9–17.6)	0.489
ANC at 0 h, ×10^3^/µL	6.49 (3.52–10.40)	6.85 (4.14–12.14)	0.260
ALC at 0 h, ×10^3^/µL	5.03 (3.05–6.84)	4.58 (2.77–6.63)	0.175

IQR, interquartile range; CPR, cardiopulmonary resuscitation; ROSC, return of spontaneous circulation; AMI, acute myocardial infarction; CHF, congestive heart failure; NLR, neutrophil-to-lymphocyte ratio; WBC, white blood cell count; ANC, absolute neutrophil count; ALC, absolute lymphocyte count. Good outcome: CPC 1–2; Poor outcome: CPC 3–5.

Of the 414 patients, 298 (72.0%) had NLR data available at 72 h and 277 (66.9%) had data at all four time points. Compared with patients included in the trajectory analysis, those with missing 72 h data were older (median 62 vs 54 years; *P* < 0.001), less likely to have a shockable rhythm (26.7% vs 43.0%; *P* = 0.003), and had a higher rate of poor neurological outcome (87.9% vs 60.7%; *P* < 0.001). These differences suggest that patients with missing data had a more severe clinical course, implying that the observed prognostic associations for NLR may be conservative estimates.

### Serial NLR dynamics

**Fig. 1:**
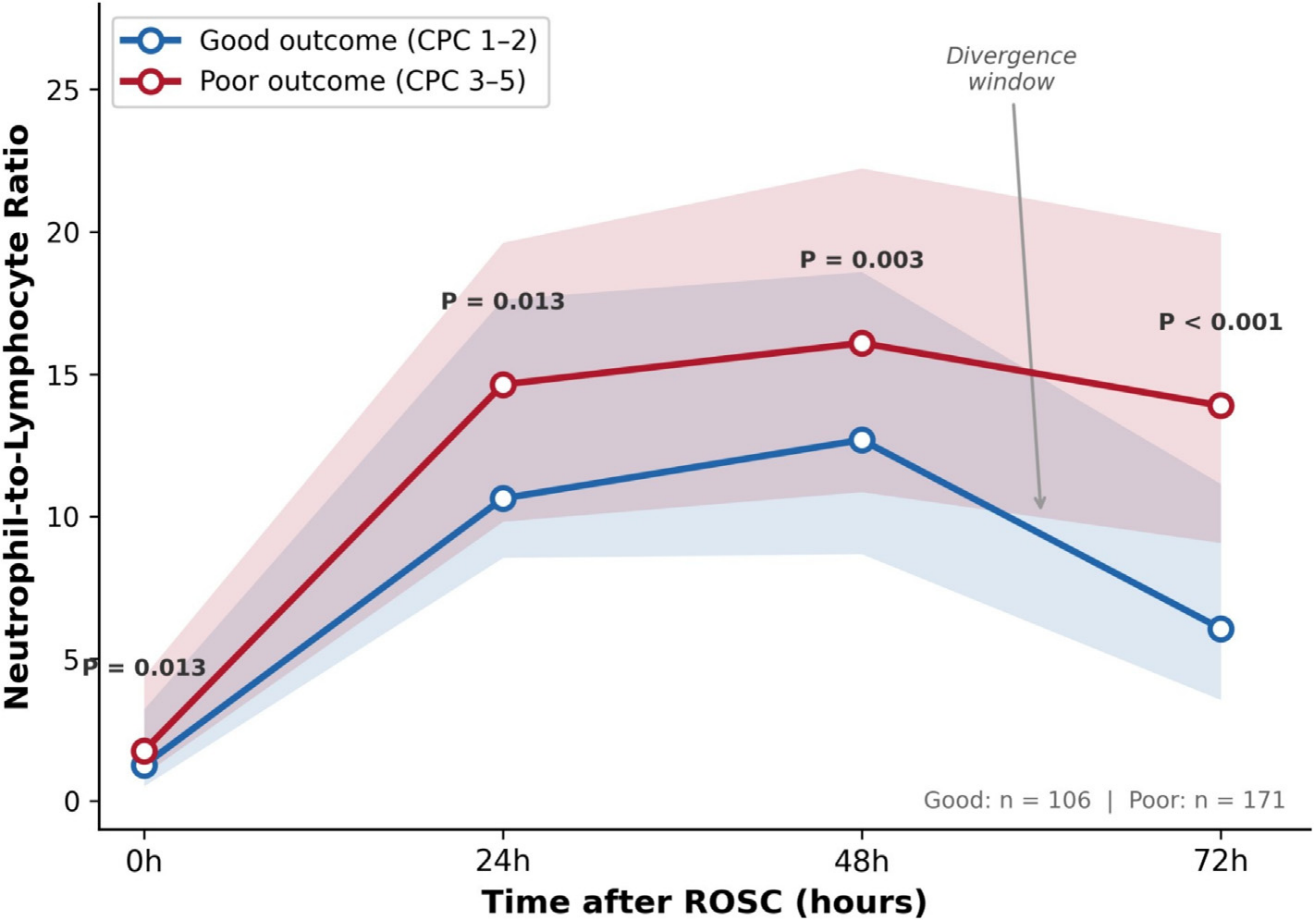
**Serial neutrophil-to-lymphocyte ratio (NLR) trajectory by neurological outcome**. Lines represent medians; shaded areas represent interquartile ranges. *P* values from Mann–Whitney *U* test are shown at each time point. NLR was comparable between groups at 0 and 24 h (both *P* > 0.05) but diverged significantly at 48 h (*P* = 0.007) and 72 h (*P* < 0.001). CPC, Cerebral Performance Category.

NLR was available in 395 patients at 0 h, 366 at 24 h, 338 at 48 h, and 298 at 72 h. At presentation (0 h), NLR was comparably low in both groups (good outcome: median 1.2 [IQR 0.6–3.2]; poor outcome: 1.6 [0.8–3.7]; *P* = 0.067). By 24 h, NLR had risen sharply in all patients, with no significant difference between groups (good: 11.0 [8.5–18.4]; poor: 13.9 [9.0–19.3]; *P* = 0.097). This uniform early rise is consistent with the expected stress response following whole-body ischemia–reperfusion (Fig. 1).

Among 277 patients with complete data at all four time points, the Friedman test confirmed significant temporal variation in NLR in both the good-outcome group (*χ*^2^ = 192.1, df = 3, *P* < 0.001) and the poor-outcome group (*χ*^2^ = 233.1, df = 3, *P* < 0.001). In a linear mixed-effects model with log-transformed NLR as the dependent variable, the group-by-time interaction was significant at 72 h (*β* = −0.37, *P* = 0.003), indicating that the temporal trajectory of NLR differed between outcome groups specifically at this time point, while the interaction terms at 24 h (*β* = 0.24, *P* = 0.054) and 48 h (*β* = 0.12, *P* = 0.312) were not significant.

**Fig. 2:**
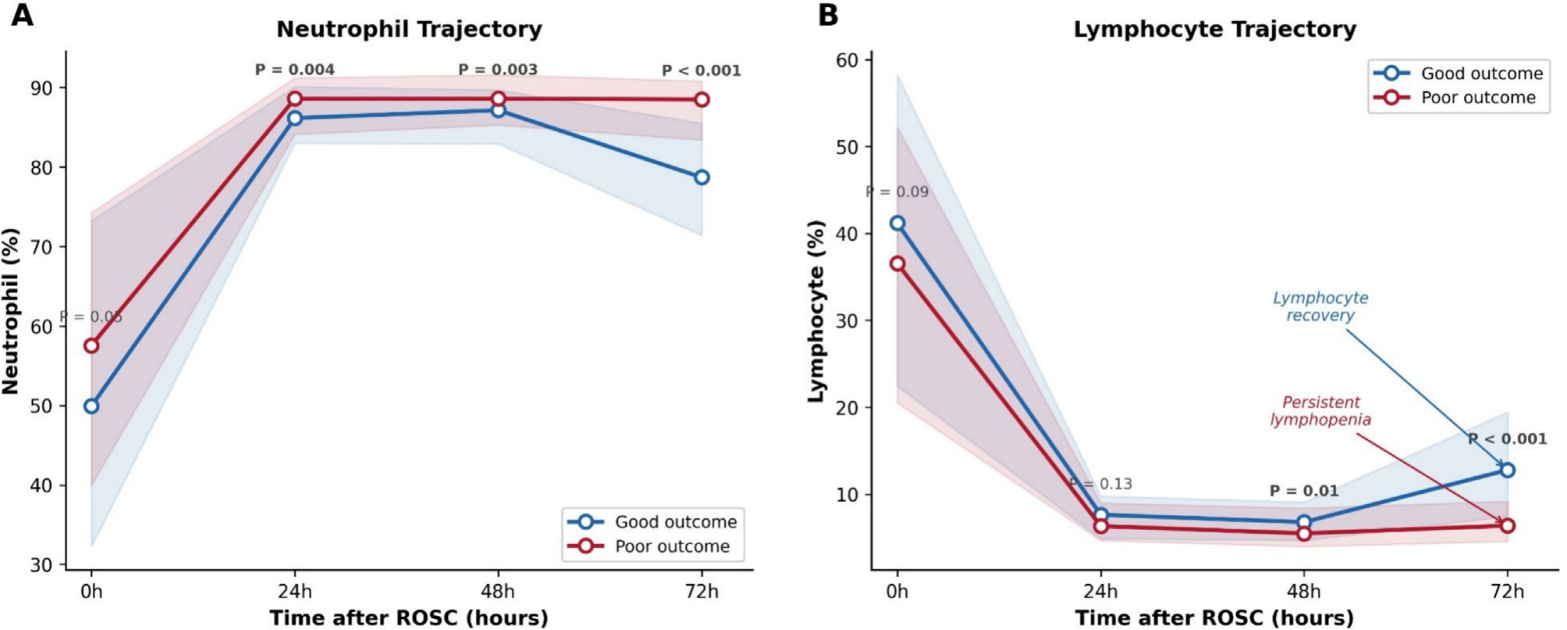
**Median trajectories of neutrophil percentage (A) and lymphocyte percentage (B) by neurological outcome**. *P* values from Mann–Whitney *U* test are displayed at each time point. The NLR divergence at 72 h was driven predominantly by lymphocyte recovery in good-outcome patients (12.8%) versus persistent lymphopenia in poor-outcome patients (6.4%).

The two groups began to diverge at 48 h (good: 12.8 [8.6–19.4]; poor: 16.1 [10.2–22.4]; *P* = 0.007), with a clear separation by 72 h (good: 6.0 [3.6–11.2]; poor: 13.9 [9.1–19.9]; *P* < 0.001; Table 2). Analysis of the individual components suggested that this divergence was driven largely by differences in lymphocyte recovery: good-outcome patients showed reconstitution from a nadir at 24 h to 12.8% at 72 h, while poor-outcome patients remained lymphopenic at 6.4% (*P* < 0.001; Fig. 2). Neutrophil percentages also differed at 72 h (good: 78.7%; poor: 88.5%; *P* < 0.001), though the relative change was less pronounced.

**Table 2:** Serial neutrophil-to-lymphocyte ratio by neurological outcome.

**Timepoint**	***n***	**Good outcome**	**Poor outcome**	***P* value**
0 h	395	1.2 (0.6–3.2)	1.6 (0.8–3.7)	0.067
24 h	366	11.0 (8.5–18.4)	13.9 (9.0–19.3)	0.097
48 h	338	12.8 (8.6–19.4)	16.1 (10.2–22.4)	0.007
72 h	298	6.0 (3.6–11.2)	13.9 (9.1–19.9)	<0.001

Values are median (interquartile range). NLR, neutrophil-to-lymphocyte ratio. *P* values from Mann–Whitney *U* test.

When analyzed using absolute lymphocyte counts (ALC), the pattern was consistent: ALC was comparable between groups at 0 h (good: median 5.03 vs poor: 4.58 × 10^3^/µL; *P* = 0.175), declined sharply by 24 h in both groups (good: 0.94 vs poor: 0.86; *P* = 0.337), and diverged at 72 h (good: 1.28 vs poor: 0.86; *P* < 0.001). Lymphopenia, defined as ALC < 1.0 × 10^3^/µL, was present in 32.5% of good-outcome patients versus 62.6% of poor-outcome patients at 72 h (*P* < 0.001). This confirms that the NLR divergence at 72 h reflects a genuine failure of lymphocyte recovery in absolute terms, not merely a shift in differential percentages.

### Prognostic performance of NLR

**Fig. 3:**
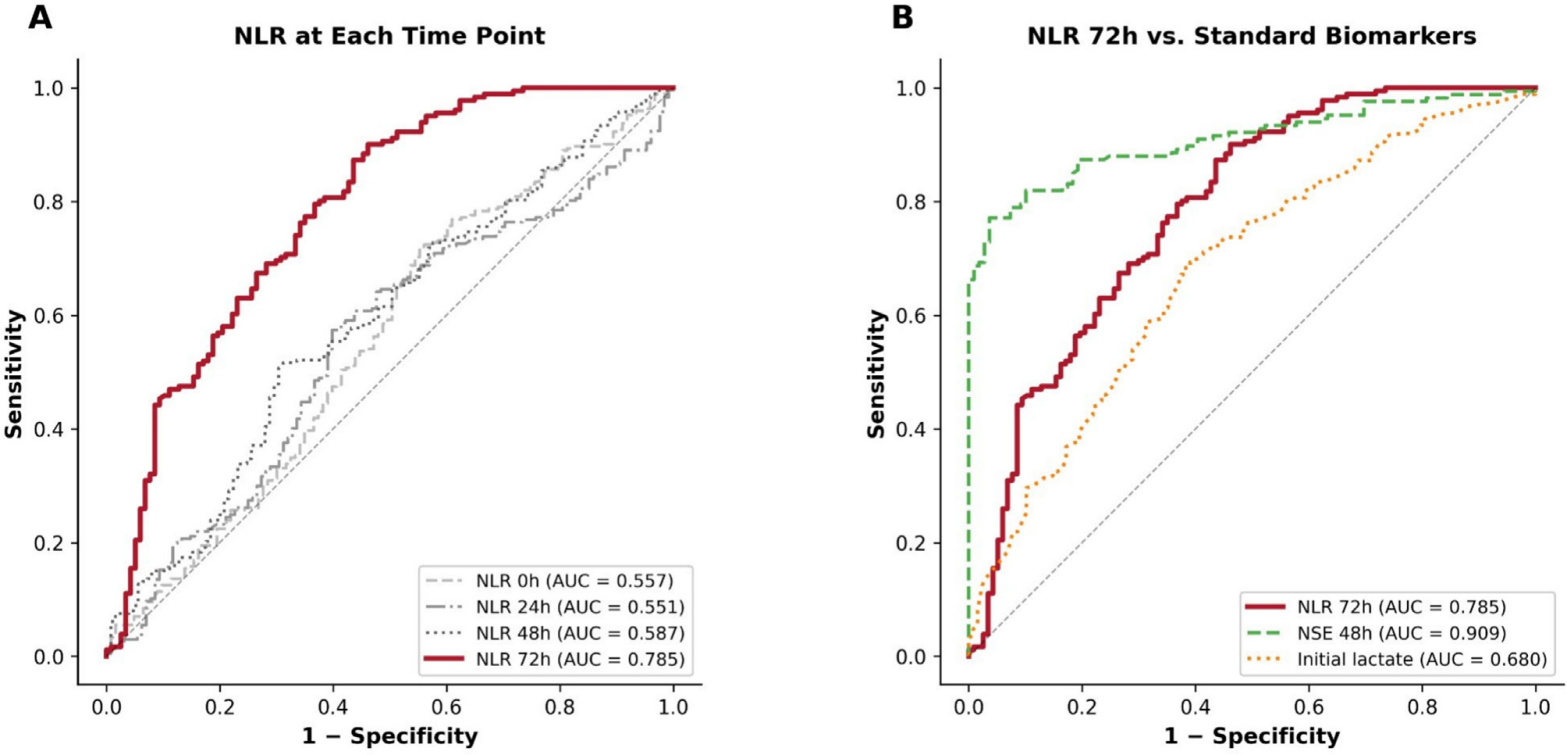
**Receiver operating characteristic curves**. (A) NLR at each time point for predicting poor neurological outcome. Discriminative ability improved progressively over time, with NLR at 72 h achieving the highest AUC of 0.785. (B) NLR at 72 h alone. AUC, area under the curve.

The discriminative ability of NLR for predicting poor neurological outcome improved progressively over time: AUC was 0.557 at 0 h, 0.551 at 24 h, 0.587 at 48 h, and 0.785 at 72 h (Fig. 3). DeLong tests confirmed that NLR at 72 h had significantly higher discriminative ability than at 0 h, 24 h, and 48 h (all *P* < 0.001), whereas differences among 0, 24, and 48 h were not statistically significant (all *P* > 0.30). At the Youden index-derived optimal cut-off of NLR > 6.3 at 72 h, sensitivity was 90.1% and specificity was 53.8%.

In multivariable logistic regression adjusted for clinical variables (age, shockable rhythm, witnessed status, bystander CPR, and time to ROSC), log-transformed NLR at 72 h remained independently associated with poor outcome (OR 5.13; 95% CI 2.88–9.14; *P* < 0.001; Table 3). A model with clinical variables alone yielded an AUC of 0.824, which improved to 0.891 with the addition of NLR at 72 h (DeLong *P* = 0.001). The continuous NRI was 0.574 (95% CI 0.341–0.807; *P* < 0.001) and the IDI was 0.071 (95% CI 0.040–0.102; *P* < 0.001).

**Table 3:** Multivariable logistic regression for poor neurological outcome at 6 months.

**Variable**	**OR**	**95% CI**	***P* value**
**Model 1: Clinical variables (AUC = 0.824)**			
Age (per year)	1.03	1.02–1.05	<0.001
Shockable rhythm	0.17	0.10–0.30	<0.001
Witnessed arrest	0.44	0.21–0.90	0.024
Bystander CPR	1.11	0.68–1.82	0.681
Time to ROSC (per min)	1.03	1.01–1.06	0.006
**Model 2: Clinical + log(NLR 72 h) (AUC = 0.891)**			
Age (per year)	1.03	1.01–1.04	0.002
Shockable rhythm	0.19	0.10–0.34	<0.001
Witnessed arrest	0.44	0.20–0.99	0.047
Bystander CPR	1.29	0.75–2.23	0.361
Time to ROSC (per min)	1.03	1.01–1.06	0.006
log(NLR 72 h)	5.13	2.88–9.14	<0.001

OR, odds ratio; CI, confidence interval; AUC, area under the receiver operating characteristic curve; NLR, neutrophil-to-lymphocyte ratio; CPR, cardiopulmonary resuscitation; ROSC, return of spontaneous circulation. Model based on *n* = 271 patients with complete data.

In the subset of 220 patients with both NLR at 72 h and NSE at 48 h available, a model with clinical variables plus NSE at 48 h yielded an AUC of 0.964. Adding NLR at 72 h produced a marginal improvement (AUC 0.967; DeLong *P* = 0.825). In the combined model, NSE remained the dominant predictor (OR 15.84; 95% CI 6.27–39.98; *P* < 0.001), while log-transformed NLR at 72 h showed a borderline independent association (OR 2.06; 95% CI 1.00–4.26; *P* = 0.050). The continuous NRI for adding NLR to the clinical-plus-NSE model was 0.515.

### NLR trajectory patterns

**Fig. 4:**
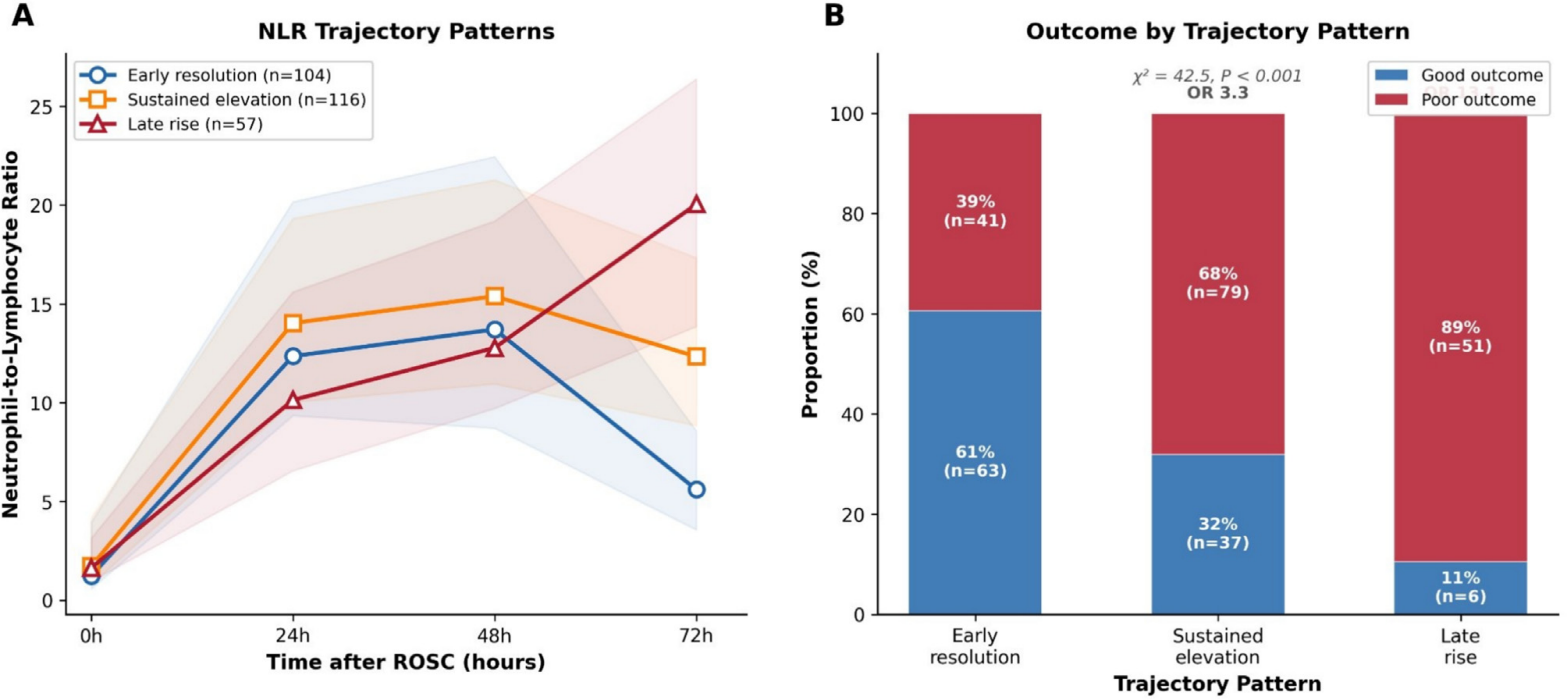
**NLR trajectory classification and outcomes**. (A) Median NLR trajectories for three classified patterns: early resolution (*n* = 104), sustained elevation (*n* = 116), and late rise (*n* = 57). (B) Proportion of good versus poor neurological outcomes within each trajectory group. OR, odds ratio versus early resolution group.

Among 277 patients with complete four-time-point data, three trajectory patterns were identified (Fig. 4A). In the early resolution group (*n* = 104, 37.5%), NLR peaked at 24–48 h and then declined, with 60.6% achieving good outcomes. In the sustained elevation group (*n* = 114, 41.2%), NLR remained elevated through 72 h, with 32.5% achieving good outcomes (OR 3.3 versus early resolution; 95% CI 1.9–5.7). In the late rise group (*n* = 59, 21.3%), NLR continued to increase at 72 h; in this group, 89.5% had poor outcomes and 10.5% achieved good outcomes (OR 13.1; 95% CI 5.1–33.1; Fig. 4B). After adjustment for age, shockable rhythm, witnessed arrest, bystander CPR, and time to ROSC, the adjusted odds ratios were 4.33 (95% CI 1.99–9.42; *P* < 0.001) for sustained elevation and 22.56 (95% CI 6.16–82.62; *P* < 0.001) for late rise (Supplementary Table S1). The distribution of outcomes differed significantly across trajectory groups (chi-square = 42.5; *P* < 0.001) (Table 4).

**Table 4:** Neurological outcomes according to NLR trajectory pattern.

**Trajectory pattern**	***n* (%)**	**Good outcome *n* (%)**	**Poor outcome *n* (%)**	**OR**	**95% CI**
Early resolution	104 (37.5)	63 (60.6)	41 (39.4)	Ref.	–
Sustained elevation	114 (41.2)	37 (32.5)	77 (67.5)	3.3	1.9–5.7
Late rise	59 (21.3)	6 (10.2)	53 (89.8)	13.1	5.1–33.1

OR, odds ratio; CI, confidence interval. Trajectory classification based on 277 patients with NLR data at all four time points (0, 24, 48, 72 h). Chi-square = 42.5, *P* < 0.001. Adjusted ORs (for age, shockable rhythm, witnessed arrest, bystander CPR, and time to ROSC): sustained elevation 4.33 (95% CI 1.99–9.42), late rise 22.56 (95% CI 6.16–82.62); see Supplementary Table S1.

Baseline characteristics differed across trajectory groups (Supplementary Table S1). The late rise group was older (median 62 years), had a lower proportion of shockable rhythms (23.7%), and was less likely to have a cardiac etiology (42.4%) compared with the early resolution group (median 48 years, 52.9% shockable, 70.2% cardiac; all *P* < 0.01).

### Sensitivity analyses

The trajectory classification was robust across different decline thresholds (25%, 50%, 75%; *P* < 0.001 for all; Supplementary Fig. S2). The late rise group consistently showed the highest proportion of poor outcomes (89–90%) regardless of the cutoff used.

In a subgroup analysis by TTM target temperature, NLR at 72 h maintained its discriminative ability in both the 33°C group (*n* = 268; AUC 0.789) and the 36°C group (*n* = 18; AUC 0.922), though the latter subgroup was small and should be interpreted with caution.

## Discussion

In this single-center cohort of OHCA survivors treated with TTM, we observed that the temporal trajectory of NLR over the first 72 h after ROSC was associated with neurological outcomes. NLR did not differentiate between outcome groups in the early post-arrest period (0–24 h), but diverged by 72 h, with an AUC of 0.785. This divergence appeared to be driven largely by a failure of lymphocyte recovery in patients with poor outcomes.

A linear mixed-effects model confirmed that the group-by-time interaction was significant specifically at the 72 h time point (*P* = 0.003), providing formal statistical evidence that the NLR trajectory—not merely the 72 h value alone—differs between outcome groups.

We tentatively use the term “immune reconstitution failure” to describe this pattern, drawing an analogy from sepsis research where persistent lymphopenia after an initial insult has been associated with impaired adaptive immunity and worse clinical outcomes.^17^, ^18^, ^19^ Importantly, analysis of absolute lymphocyte counts confirmed this finding: good-outcome patients recovered from a median ALC of 0.94 at 24 h to 1.28 × 10^3^/µL at 72 h (above the standard lymphopenia threshold), while poor-outcome patients remained lymphopenic (ALC 0.86 at 72 h). We acknowledge that this terminology is speculative in the cardiac arrest setting, as our observational design cannot establish a causal relationship between lymphocyte recovery failure and neurological injury.

Our findings build on previous single-time-point NLR studies in cardiac arrest. Kim et al. reported that NLR ≥ 6 at 72 h was associated with poor outcomes (OR 7.4) in 216 OHCA patients,^14^ and Mortberg et al. showed that NLR changes during TTM predicted survival.^16^ and Miyatake et al. linked low lymphocyte counts during therapeutic hypothermia to poor neurological outcomes.^20^ Our data suggest that the prognostic value may lie not in the absolute NLR value itself, but in whether NLR resolves after the initial stress response.

The biological mechanism underlying our observations may relate to the immune dysregulation described after cardiac arrest. Whole-body ischemia–reperfusion triggers a catecholamine-driven stress response that causes demargination of neutrophils and redistribution of lymphocytes to lymphoid organs.^21^, ^22^ In patients who subsequently recover, lymphocyte counts begin to reconstitute by 48–72 h.^17^, ^19^ In those with severe hypoxic–ischemic brain injury, persistent sympathetic activation and hypothalamic–pituitary–adrenal axis dysregulation may sustain the lymphopenic state,^23^, ^24^ with parallel disturbances reported in other immune lineages such as monocyte subsets.^25^

The combined model analysis suggests that NLR at 72 h provides limited incremental discrimination beyond NSE at 48 h (AUC 0.964 vs 0.967; *P* = 0.825), which is expected given the exceptional performance of NSE in this cohort. However, the borderline independent association of NLR in the combined model (*P* = 0.050) and the continuous NRI of 0.515 suggest that NLR may contribute to individual-level reclassification even when NSE is available. More importantly, the principal clinical role of NLR trajectory is not to compete with NSE but to serve as an accessible surrogate in settings where NSE assays are unavailable or delayed—a situation that remains common in many healthcare systems worldwide. The trajectory pattern itself (resolution vs persistence vs escalation) also conveys qualitative prognostic information that a single NSE value cannot capture.

We chose to compare NLR at 72 h (rather than at 48 h) with NSE at 48 h because 72 h was the time point at which NLR showed its peak discriminative ability (AUC 0.785 vs 0.587 at 48 h). The comparison was intended to benchmark the best-performing NLR measurement against the established standard.

A practical consideration is that the strongest prognostic signal emerges at 72 h, whereas clinicians may need prognostic information earlier. However, current ERC/ESICM 2025 guidelines recommend deferring formal neuroprognostication to at least 72 h after ROSC,^7^ which aligns with the optimal timing of NLR assessment. The limited discrimination of NLR at 0 and 24 h (AUC 0.557 and 0.551) is informative: the uniformly elevated early NLR reflects a shared stress response that does not yet differentiate between outcome groups. In practice, the serial monitoring approach may offer interim signals—a patient whose NLR is already declining at 48 h is more likely to follow the early resolution pattern. Future studies should explore whether the rate of NLR decline between 24 and 48 h could provide earlier prognostic information.

The influence of TTM on the inflammatory and immune response warrants discussion. Hypothermia is known to modulate leukocyte trafficking: the induction and maintenance phases of TTM (typically the first 24–36 h after ROSC) may suppress neutrophil activation and delay lymphocyte redistribution, contributing to the uniformly elevated NLR observed in both outcome groups at 24 h. During the rewarming phase (approximately 36–48 h) and the subsequent normothermic period (48–72 h), the restraint on immune cell dynamics is released, potentially unmasking the underlying difference in immune reconstitution capacity between outcome groups. This timeline is consistent with our observation that NLR diverges primarily after 48 h. In our cohort, both 33°C and 36°C protocols were used, with 33°C predominating (91.1%). NLR at 72 h maintained its discriminative ability in both temperature subgroups (33°C: AUC 0.789; 36°C: AUC 0.922), although the small number of 36°C patients (*n* = 18) limits comparison between protocols. Future studies with balanced TTM groups should systematically examine how different temperature targets (including active normothermia) influence the kinetics of NLR trajectory.

From a practical standpoint, NLR trajectory may serve as a freely available complement to established prognostic tools. The principal advantage of NLR lies in its accessibility: it requires no additional blood draw, no specialized assay, and is available in any hospital with a hematology analyzer.

### Limitations

Several limitations should be considered. First, this is a single-center retrospective study, which limits generalizability. Second, NLR was calculated from percentage differentials rather than absolute counts, which may be influenced by total white blood cell count variations. Third, medications that affect leukocyte counts, particularly corticosteroids and catecholamines, were not systematically recorded in our registry and could not be incorporated as covariates.

However, we performed a supplementary analysis using absolute lymphocyte counts that confirmed the percentage-based findings. Moreover, because NLR calculated from percentages (neutrophil%/lymphocyte%) is mathematically identical to ANC/ALC (the total WBC cancels in the ratio), the NLR values themselves are not affected by total white blood cell count variations.

Our registry did not systematically capture ICU complications such as nosocomial infections, corticosteroid doses, or vasopressor requirements. While our multivariable model adjusts for the major clinical predictors recommended by international guidelines, residual confounding from unmeasured variables cannot be excluded. Initial CRP and procalcitonin values were available but were not included as covariates because they reflect the same systemic inflammatory process that NLR captures, and their inclusion would introduce collinearity. Future prospective studies should systematically record medication exposures and ICU complications.

Fourth, approximately 28% of patients lacked 72 h NLR data. These patients had more severe baseline profiles and worse outcomes, indicating that the missingness was not random but related to clinical severity. Our findings therefore apply specifically to patients who survive to 72 h with available laboratory data, and the observed associations may represent conservative estimates.

Fifth, the TTM protocol evolved over the 15-year study period, with both 33°C and 36°C used from 2014 onward. Although 33°C remained the predominant target (91.1%), the small 36°C subgroup (*n* = 27) limits our ability to draw conclusions about protocol-specific effects on NLR. Sensitivity analysis showed consistent NLR discrimination in both temperature subgroups.

Sixth, the trajectory classification cut-off (50% decline from peak) was empirically defined. Although data-driven approaches such as GBTM or LCGA could provide more objective classification, our sample size (*n* = 277) may be insufficient for stable latent class estimation. Sensitivity analyses using 25% and 75% thresholds showed consistent results. Finally, the absence of WLST in our center, while protecting against self-fulfilling prophecy, may limit applicability to settings where WLST is commonly practiced.

## Ethical approval

This study was approved by the Institutional Review Board of the Catholic University of Korea, Seoul St. Mary’s Hospital (KC23RISI0264; approved 21 April 2023). The requirement for informed consent was waived given the retrospective nature of the analysis.

## CRediT authorship contribution statement

**Kyungman Cha:** Writing – original draft, Formal analysis, Conceptualization. **Sang Hoon Oh:** Project administration. **Jee Yong Lim:** Writing – review & editing, Writing – original draft, Supervision, Project administration, Conceptualization.

## Funding

This study was supported by the Research Fund of Seoul St. Mary’s Hospital, The Catholic University of Korea (Project No. ZC26RISI0146).

## Declaration of competing interest

The authors declare that they have no known competing financial interests or personal relationships that could have appeared to influence the work reported in this paper.

## Appendix A Supplementary material

Supplementary Data 1 Including a correlation analysis with neuron-specific enolase (Fig. S1), sensitivity analyses for trajectory classification thresholds (Fig. S2), and baseline characteristics by trajectory pattern (Table S1).
