# Supplementary material for: Trajectory of serial neutrophil-to-lymphocyte ratio predicts neurological outcome after out-of-hospital cardiac arrest
Source: Resusc Plus. 2026 Apr 21;29:101337. doi: 10.1016/j.resplu.2026.101337 (PMC13158605; doi:10.1016/j.resplu.2026.101337)
Supplement: Supplementary Data 1 — Including a correlation analysis with neuron-specific enolase (Fig. S1), sensitivity analyses for trajectory classification thresholds (Fig. S2), and baseline characteristics by trajectory pattern (Table S1). [file mmc1.docx]

**Supplementary Table S1.** Baseline characteristics and neurological outcomes according to NLR trajectory pattern (n = 277 patients with complete four-time-point data)

| **Variable** | **Early resolution**  **(n = 104)** | **Sustained elevation**  **(n = 114)** | **Late rise**  **(n = 59)** | **P value** |
| --- | --- | --- | --- | --- |
| Age, years, median (IQR) | 47.6 (35.5–57.2) | 57.5 (46.9–67.0) | 62.0 (47.5–71.0) | <0.001 |
| Male sex, n (%) | 74 (71.2) | 84 (73.7) | 43 (72.9) | 0.908 |
| Cardiac etiology, n (%) | 73 (70.2) | 71 (62.3) | 25 (42.4) | 0.002 |
| Shockable rhythm, n (%) | 55 (52.9) | 50 (43.9) | 14 (23.7) | 0.001 |
| Witnessed arrest, n (%) | 74 (71.2) | 82 (71.9) | 36 (61.0) | 0.295 |
| Bystander CPR, n (%) | 74 (71.2) | 77 (67.5) | 33 (55.9) | 0.134 |
| Time to ROSC, min, median (IQR) | 2.0 (0.0–8.5) | 2.0 (0.0–5.0) | 5.0 (0.0–10.0) | 0.116 |
| NLR at 0 h, median (IQR) | 1.2 (0.6–3.9) | 1.7 (0.8–4.0) | 1.9 (1.0–3.4) | 0.126 |
| NLR at 24 h, median (IQR) | 12.4 (9.3–20.2) | 14.0 (10.1–19.5) | 10.1 (6.8–15.7) | 0.010 |
| NLR at 48 h, median (IQR) | 13.7 (8.7–22.5) | 15.7 (10.9–21.8) | 12.8 (9.7–18.6) | 0.065 |
| NLR at 72 h, median (IQR) | 5.6 (3.6–8.6) | 12.2 (8.8–17.3) | 20.0 (13.7–26.0) | <0.001 |
| Good outcome, n (%) | 63 (60.6) | 37 (32.5) | 6 (10.2) | <0.001 |
| Unadjusted OR (95% CI) | Ref. | 3.3 (1.9–5.7) | 13.1 (5.1–33.1) | — |
| Adjusted OR (95% CI)* | Ref. | 4.33 (1.99–9.42) | 22.56 (6.16–82.62) | — |

*OR, odds ratio; CI, confidence interval; IQR, interquartile range; CPR, cardiopulmonary resuscitation; ROSC, return of spontaneous circulation; NLR, neutrophil-to-lymphocyte ratio. Continuous variables compared using the Kruskal–Wallis test; categorical variables compared using the chi-square test.*

**Adjusted for age, initial shockable rhythm, witnessed arrest, bystander CPR, and time to ROSC. Reference category: early resolution.*


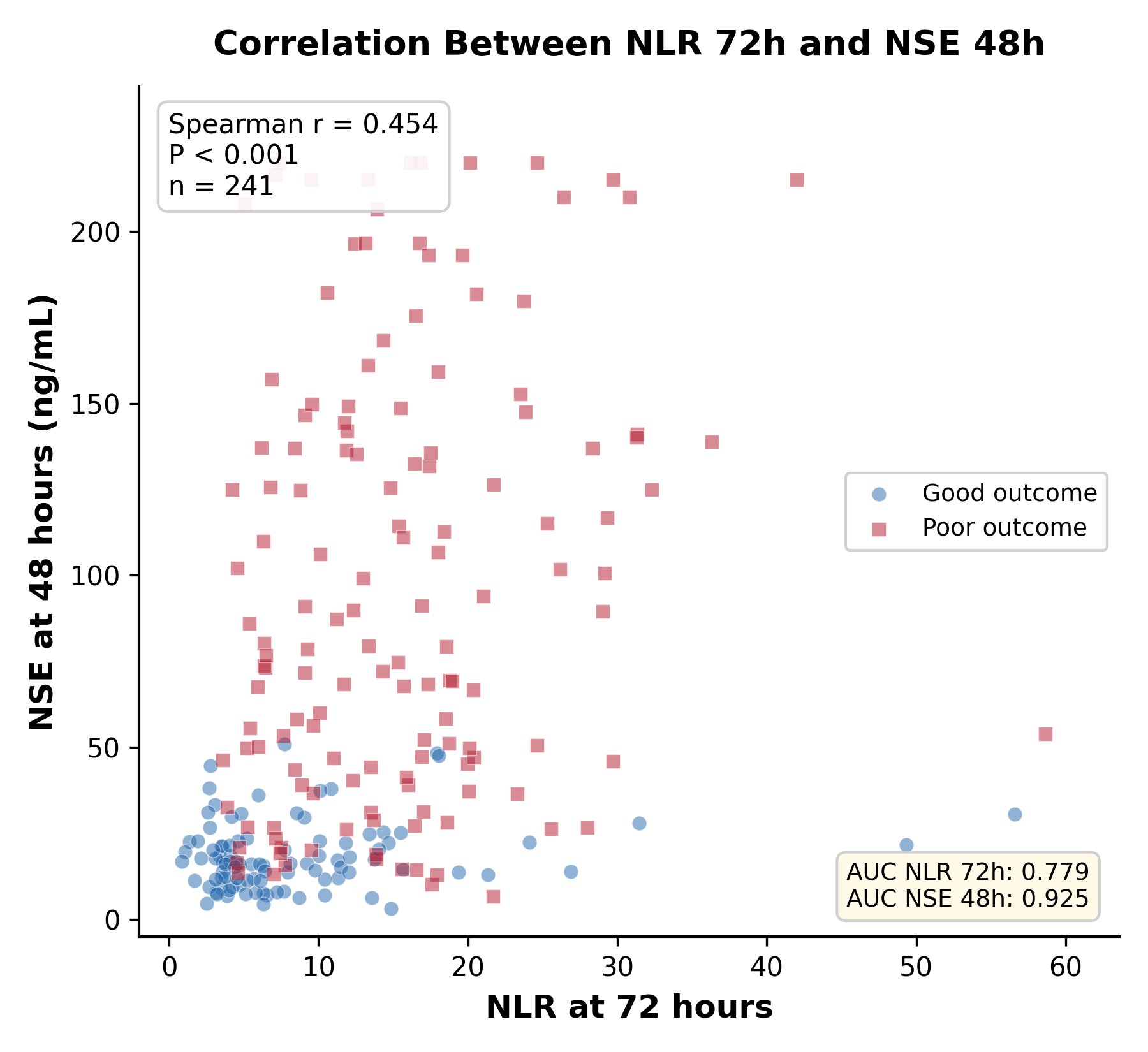


*Supplementary Fig. S1. Scatter plot showing the correlation between NLR at 72 h and neuron-specific enolase (NSE) at 48 h in 241 patients with both biomarkers available. The two markers showed moderate positive correlation (Spearman r = 0.454; P < 0.001). AUC for NSE 48 h (0.925) exceeded that for NLR 72 h (0.779).*


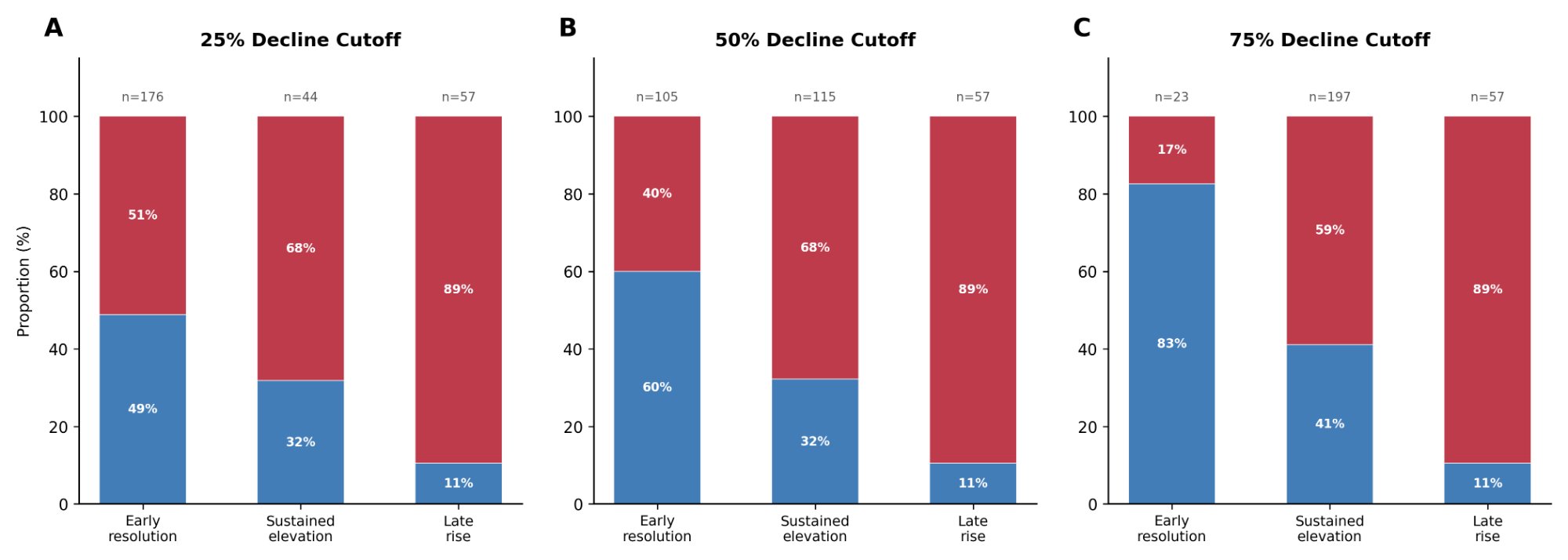


*Supplementary Fig. S2. Sensitivity analysis of trajectory classification using different decline thresholds: 25% (A), 50% (B, primary analysis), and 75% (C). The association between trajectory pattern and neurological outcome remained significant across all thresholds (P < 0.001 for all), with the late rise group consistently showing the highest proportion of poor outcomes (89–90%).*
